# Relationship between physical activity during pregnancy and maternal health outcomes: evidence from the MAASTHI cohort study in Bengaluru, India

**DOI:** 10.3389/fspor.2025.1265929

**Published:** 2025-01-30

**Authors:** Yamuna Ana, Floor A. van den Brand, Onno C. P. van Schayck, Giridhara R. Babu

**Affiliations:** ^1^ Indian Institute of Public Health-Bengaluru, Public Health Foundation of India (PHFI), Bengaluru, India; ^2^ Department of Family Medicine, Care and Public Health Research Institute (CAPHRI), Maastricht University, Maastricht, Limburg, Netherlands; ^3^ Department of Population Medicine, College of Medicine, QU Health, Qatar University, Doha, Qatar

**Keywords:** physical activity, C-section delivery, maternal health outcomes, life course approach, cohort study

## Abstract

**Background:**

Sedentary behavior is one of the major modifiable behavioral risk factors for non-communicable diseases. Physical activity (PA) is crucial during pregnancy but pregnant women may become sedentary, leading to adverse health outcomes. Our study aimed to explore the association between social support and PA levels during pregnancy and the relationship between sedentary behavior and adverse pregnancy health outcomes including delivery.

**Methods:**

The study used a validated physical activity questionnaire to assess the physical activity levels of pregnant women. We collected detailed sociodemographic information, pregnancy characteristics, assessed social support. We assessed presence of depressive symptoms and conducted oral glucose tolerance tests, hemoglobin and blood pressure assessments, anthropometric measurements, and collected delivery details. We used linear logistic regression to assess the association between a continuous measure of physical activity level and maternal outcomes and performed multivariable logistic regression analysis to understand the association between sedentary behavior and maternal health outcomes and mode of delivery after adjusting for potential confounders.

**Results:**

We interviewed 2,424 eligible pregnant women at baseline and 1,317 were considered in the final analysis after excluding those who missed follow-ups. We observed that one unit increase in physical activity level was associated with reduced prenatal depressive symptoms (*β* = −6.36, *p* < 0.001), fasting (*β* = 2.06, *p* = 0.04), and postprandial blood sugar levels (*β* = −0.99, *p* = 0.01), respectively. Pregnant women who had good social support tended to engage in higher levels of activity. In addition, women who engaged in sedentary behavior during pregnancy were 1.07 times more likely to be obese and 4.32 times more likely to have elective cesarean section (C-section) delivery than those who engaged in moderate activity.

**Conclusion:**

The study found that physical activity during pregnancy has several beneficial effects on maternal prenatal health outcomes, including a reduced risk of obesity and C-section delivery, lower blood glucose levels, and improved mental health. Therefore, it is essential to adhere to the recommended guidelines for physical activity during pregnancy. Healthcare providers and policymakers in India should consider promoting physical activity as part of comprehensive routine prenatal care.

## Introduction

Sedentary behavior during pregnancy could impose significant risks to maternal wellbeing and nearly two-thirds of pregnant women have been found to be sedentary (1). Physical activity (PA) helps women carry out daily activities with more liveliness and can reduce the risk of diseases caused by physical inactivity (2). Being physically active has beneficial effects for pregnant women who were sedentary before pregnancy (3). It is suggested that pregnant and postpartum women without complications should accomplish at least 150 min of moderate-intensity PA per week (4–7), but only approximately one in ten pregnant women meet the suggested guidelines in India (8). Healthy weight and higher educational attainment are positively associated with PA (9), depending on socio-demographics and maternal health conditions. Physically active pregnant women are less likely to have adverse maternal health outcomes such as cesarean section (C-section) delivery (10), gestational diabetes mellitus (GDM) (11), hypertension, obesity (12), and maternal depression (13).

Globally, almost one in five women will give birth via C-section delivery (14). In Karnataka, according to the National Family Health Survey (NFHS)-5, 31.5% of births are delivered by C-section and the prevalence has increased compared to NFHS-4 (23.6%) which could be due to several medical conditions, including the preference of women. The NFHS is a household survey that provides state and national information for India on women and child health indicators. Moreover, C-section rates are higher in urban areas than in rural areas (15). Many factors contribute to adverse delivery outcomes, and most prominently pregnancy conditions such as diabetes, hypertension, anemia, obesity, and sedentary behavior. Efforts to decrease overall C-section rates are important, as it is associated with several adverse outcomes in women and children, including an association with excess weight and reduced linear growth at 1 year of age in children in low- and middle-income countries (LMICs) (16). There are several mechanisms which explain such an association. Among these, the leading hypothesis is that there is a relationship between sedentary behavior and birth via C-section delivery. Sedentary behavior during pregnancy can increase the likelihood of C-section delivery through several mechanisms. These include excessive weight gain during pregnancy, which can result in a larger fetus that may be difficult to deliver vaginally, and poor muscle strength and endurance, which can make it more difficult for women to push during labor. In addition, sedentary behavior can lead to poor cardiovascular fitness, increasing the risk of fatigue and exhaustion during labor, which can also increase the likelihood of C-section delivery. Finally, sedentary behavior can increase the risk of developing GDM and hypertension, which are linked to a higher risk of C-section delivery (17). Furthermore, evidence shows nearly two-thirds of the sedentary women were obese at the end of pregnancy (18), and a higher level of PA in the first trimester of pregnancy reduced the risk of GDM by 20% (19). Pregnant women who engage in sedentary behavior had a three times higher chance of developing postpartum depressive symptoms (20).

Poor social support is a vital risk factor for poor maternal health outcomes. Higher social support is associated with an improved level of PA (21). Pregnant women who have good family and social support tend to adopt a healthy lifestyle (22). Pregnant women with improved PA were found to have better cardiovascular function and reduced risk of GDM and hypertension (23). However, the extent to which PA during pregnancy influences the mode of delivery is still a topic of debate. In addition, this study examined several adverse pregnancy outcomes, which pose significant risks to both pregnant women and their children. Furthermore, investigating the association between modifiable risk factors and these outcomes could aid in the adoption of a comprehensive health strategy. Given that no such study has been done in South India in a birth cohort study, we hypothesized that there is a relationship between social support and the level of PA among pregnant women and that the level of PA has an association with adverse pregnancy health outcomes. Our study aimed to explore the association between social support and PA levels (PAL) during pregnancy and the relationship between sedentary behavior during pregnancy and adverse pregnancy health outcomes including delivery. The outcomes examined were delivery type, depressive symptoms, hypertension, GDM, and maternal obesity.

## Materials and methods

### Study setting

This study was conducted within a longitudinal cohort study entitled “Maternal Antecedents of Adiposity and Studying the Transgenerational Role of Hyperglycemia and Insulin (MAASTHI),” and the protocol of this has been previously published (24, 25). We recruited pregnant women from public hospitals in urban Bengaluru in the gestational age range of 14–36 weeks after obtaining written informed consent. The inclusion criteria included pregnant women between the ages of 18 and 45 years who were less than 36 weeks of gestation, planned to deliver in the study location, and were available for follow-up soon after delivery. The exclusion criteria included a history of diabetes, Hepatitis B infection, and human immunodeficiency virus (HIV) positivity. The study period was from 2016 to 2021.

Assuming an incidence of cesarean delivery of 7% among mothers with moderate to vigorous physical activity levels during pregnancy (26) and a relative risk of 1.8 in the sedentary behavior group, our estimated sample size for 80% power to detect a difference at a 95% confidence level was 878. Further assuming a follow-up of up to 50%, the calculated sample size was 1,317.

### Data collection

**Figure 1:**
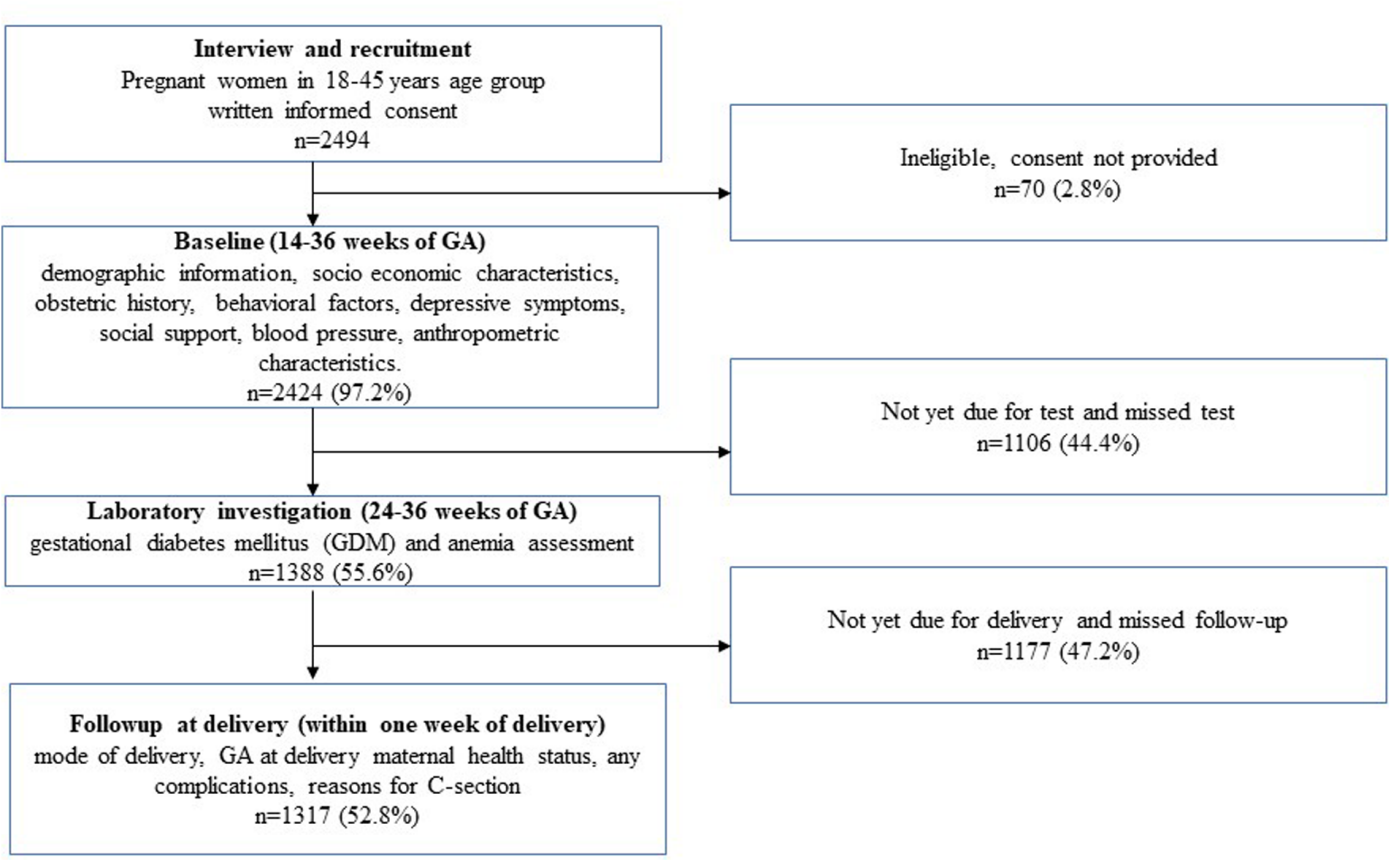
Schematic flow of assessments from recruitment to follow-ups for assessing the relationship between sedentary behavior during pregnancy and adverse maternal and delivery health outcomes.

Trained research assistants interviewed the pregnant women at the public hospitals maintaining privacy during the interviews. We collected demographic information, socioeconomic characteristics, obstetric history, behavioral factors, depressive symptoms, and social support information in addition to measuring blood pressure (BP), GDM status, and anthropometric characteristics during pregnancy. We followed up with the participants after delivery, within 1 week postpartum, and obtained follow-up data, including information on the mode of delivery, maternal health status, and any complications during delivery (Figure 1). We executed data collection using a designed application on Android tablets, with rigorous actions applied to safeguard the collected data such as Android-based tablets for data collection and real-time monitoring of the data, and a website was developed for the project to maintain an audit trail of all the data entries made in the app (27).

### Exposure assessment

While recruiting the pregnant women, trained research assistants assessed PA using the physical activity questionnaire (28). The correlation of this questionnaire with that of the accelerometer was 0.28; *p* = 0.01 and with 24 h PA dairy was *p* = 0.006 and this questionnaire was validated for use in Bengaluru, India (28). All the research assistants were trained on this questionnaire at St. Johns Research Institute, Bengaluru, and a pilot cohort study was conducted to pre-test the questionnaire and assess the feasibility of conducting the study (29). The questionnaire covers various domains to capture information on sedentary, moderate, and vigorous activity. The participant's PA data were collected by recording the frequency, duration, and type of activity in each domain, including work, leisure time, and transportation. The duration of each activity was recorded in minutes per day. PAL was calculated using parameters such as the duration of each activity, its energy cost, and total duration of activity using the formula sum of (time × energy cost of each activity)/total duration of activity. This is also termed energy requirement, expressed as a multiple of the 24-hour basal metabolic rate (BMR). To classify the level of PA, we used PAL cutoffs. Participants were classified as sedentary if their PAL value was 1.40–1.69, moderately active if their PAL value was between 1.70 and 1.99, and vigorously active if their PAL value was 2.00–2.40 (30).

### Outcome assessment

We assessed prenatal depressive symptoms using the Edinburgh Postnatal Depression Scale (EPDS) during the baseline recruitment (31), which is the most widely used screening tool for the detection of perinatal depression (32). The questionnaire was validated to assess prenatal depression for use in Karnataka, India (sensitivity = 100%, specificity = 84.90%, and area under the curve (AUC) = 0.95) (31). The pregnant women had to choose a response from the list for each question, considering the previous week. The upper limit of the EPDS score is 30 and according to the previous evidence, women with EPDS scores above 13 were categorized as having prenatal depressive symptoms (33). We counseled the women found to have depressive symptoms and referred them to the clinical psychiatrists at the public health facilities. We explained the study to them and the questionnaire was used to assess depressive symptoms and a referral mechanism was established with the hospital staff. We measured social support using a validated social support questionnaire which was developed at St. John's Research Institute, Bengaluru, and as there are no defined cutoffs for this score, median cutoffs were considered for categorizing the scores into poor (0–24) and good (>24) social support scores (34). The instrument had an alpha coefficient of 0.88 (35). This questionnaire covers a broad range of support aspects such as emotional, instrumental, informational, and appraisal support. It captures data on the availability of people in the pregnant woman’s social environment who can look after the children, take them to the hospital during an emergency; provide something that is needed during emergencies; give advice; provide information, care, and support; talk about personal topics; appreciate good work; and respect personal qualities.

We conducted a 2 h 75 g oral glucose tolerance test (OGTT) to identify GDM and the test was conducted between 24 and 36 weeks of gestational age after overnight fasting for at least 8 h. At the study centers, a trained phlebotomist collected 2 ml blood both during fasting and 2 h postprandial for glucose analysis, and the test was conducted at an accredited laboratory. Using the World Health Organization (WHO) diagnostic criteria, GDM cases were identified if the fasting blood sugar (FBS) was ≥92 mg/dl, or 2 h postprandial blood sugar (PPBS) was ≥153 mg/dl (36). To measure obesity, we considered the sum of skinfold thickness (SSFT) by taking the average value of bicep, tricep, and subscapular skinfold thickness as measured using calipers (Holtain, UK). This measure has a greater diagnostic capability for obesity (37) and an SSFT >90th percentile cutoff was used to define obesity (38, 39). To measure the mid-upper arm circumference (MUAC), Chasmors body circumference tape was used and two measurement readings to the nearest 0.1 mm were documented. Trained research assistants who are certified annually at the St. Johns Research Institute, Bengaluru, conducted all the anthropometric measurements. We assessed the mother's BP using an automated digital device (Omron Digital BP measuring device) and documented two readings of systolic blood pressure (SBP) and diastolic blood pressure (DBP). We used the Joint National Committee 8 (JNC 8) criteria to diagnose hypertension (40). Pregnant women diagnosed with GDM, hypertension, or obesity were referred to the gynecologists at the study hospitals. Since the study was set up at public health facilities and the research team worked closely with the maternal and child health wings of the hospitals, this paved the way to refer the pregnant women directly to the gynecologists for further management. When requested by the doctors, the research team also performed a retest of the pregnant women for GDM to confirm the diagnosis. During follow-up after delivery, the mode of delivery of the women was documented by referring to their medical records. Vaginal delivery types included spontaneous and assisted vaginal delivery. C-section delivery is inclusive of both emergency and elective C-section.

### Covariates

Detailed demographic details such as the age of the pregnant women, education, and total monthly income of the family were collected during the time of the recruitment according to the prevalidated tool (41). To assess the socioeconomic status of the pregnant women, we used data on education, occupation, and income. The socioeconomic status of the participants was categorized into upper, middle, and lower class using the recent version of the Kuppuswamy scale (41). Details regarding the parity of the pregnant women were collected from their medical records and verified during the interview. The hemoglobin level of the pregnant women was measured at an accredited laboratory and if the hemoglobin level was <11 g/dL, it was categorized as anemia according to the WHO criteria (42).

### Statistical analysis

Research assistants entered the collected data into a designated application built for Android devices. The study analysis was executed using the Statistical Package for the Social Sciences (SPSS) version 23.0 (IBM Corp. Released 2015, Armonk, NY) statistical software. We measured the normality of data using the Shapiro–Wilk test before applying parametric tests and variables with significance >0.05 were considered. We used the chi-square test to test whether the exposure and outcome variables were related to each other. The effect size was measured using the independent samples *t*-test for each outcome assessed in the study and findings showed medium and small effects (Supplementary File 1). We performed a linear logistic regression to evaluate the association between the continuous PA data and each continuous measure of pregnancy characteristics. For the adjusted analysis, all the other factors are considered as confounders and are presented as correlation coefficients, 95% confidence interval, and the *p*-value. We performed a multivariable logistic regression analysis where one independent variable was assessed in association with multiple outcomes that had multiple categories. This study measured the association of sedentary behavior during pregnancy on the mode of delivery and other maternal characteristics after adjusting for the potential confounders, such as outcomes that were significant in the linear regression and from earlier evidence (43). We presented the results as the odds ratio, 95% confidence interval, and the *p*-value.

## Results

In the MAASTHI cohort, we interviewed 2,494 pregnant women and among them, 97.2% completed the baseline interviews and we included 52.8% of the participants after excluding those who did not have follow-up data (Figure 1). The study found that two-thirds of the pregnant women were 18–25 years old and 47.1% were primiparous. The study found that 7.4% of the women reported having prenatal depressive symptoms, and 6.6% engaged in sedentary behavior. The mode of delivery was recorded and 47.0% of the participants had a C-section delivery (Table 1).

**Table 1:** Descriptive characteristics of the pregnant women in the MAASTHI cohort, South India (*N* = 1,317).

Parameter	Category	*N* (%)
Age (years)	18–25	870 (66.1)
	26–35	430 (32.6)
	36–45	17 (1.3)
SES	Lower	800 (60.8)
	Middle	513 (39)
	Upper	3 (0.2)
Parity	Nullipara	574 (43.6)
	Primipara	620 (47.1)
	Multipara	123 (9.3)
Prenatal depressive symptoms	Present	97 (7.4)
Hemoglobin status	Anemia	543 (42.7)
Physical activity level	Sedentary	87 (6.6)
	Moderate	1,230 (93.4)
GDM status	GDM	190 (15.1)
Mother's SSFT	<10th	126 (9.8)
	10th–90th	1,034 (80.3)
	>90th	127 (9.9)
Height of the mother	<10th	119 (9.2)
	≥10th	1,169 (90.8)
Blood pressure	Hypertension	78 (6.1)
Mode of delivery	Normal	698 (53.0)
	Cesarean	619 (47.0)

GDM, gestational diabetes mellitus; PA, physical activity; SES, socioeconomic status; SSFT, sum of skinfold thickness.

The study found that among the pregnant women who engaged in sedentary behavior, 70.1% were in the 18–25 year age group, 59.8% had low socioeconomic status, 54.0% were nulliparous, 69.0% had low social support, 93.1% had depressive symptoms, 11.5% were obese, 19.0% had GDM, 6.9% had hypertension, and 57.5% underwent a C-section delivery, which was higher among women who were sedentary compared to women who engaged in a moderate level of activity (Table 2).

**Table 2:** Distribution of maternal and delivery characteristics based on physical activity level during pregnancy in the MAASTHI cohort, South India (*N* = 1,317).

Parameter	Category	Sedentary, *n* (%)	Moderate activity, *n* (%)	*p*-value
Age (years)	18–25	61 (70.1)	809 (65.8)	0.68
	26–35	25 (28.7)	405 (32.9)	
	36–45	1 (1.1)	16 (1.3)	
Education	Illiterate	2 (2.3)	29 (2.4)	0.82
	Primary and middle school	17 (19.5)	249 (20.3)	
	High school	43 (49.4)	548 (44.6)	
	PUC and higher	25 (28.7)	403 (32.8)	
SES	Lower	52 (59.8)	748 (60.9)	0.85
	Middle	35 (40.2)	478 (38.9)	
	Upper	0 (0)	3 (0.2)	
Parity	Nullipara	47 (54.0)	527 (42.8)	0.12
	Primipara	33 (37.9)	587 (47.7)	
	Multipara	7 (8.0)	116 (9.4)	
Prenatal depressive symptoms	Absent	6 (6.9)	91 (7.4)	1.0
	Present	81 (93.1)	1,139 (92.6)	
Social support score	0–24	60 (69.0)	780 (63.4)	0.30
	>24	27 (31.0)	450 (36.6)	
Prenatal SSFT (percentile)	<10th	5 (5.7)	123 (10.0)	0.39
	10–90th	72 (82.8)	987 (80.3)	
	>90th	10 (11.5)	119 (9.7)	
Hemoglobin level	Normal	45 (53.6)	683 (57.5)	0.49
	Anemia	39 (46.4)	504 (42.5)	
GDM status	No	68 (81.0)	1,000 (85.2)	0.34
	Yes	16 (19.0)	174 (14.8)	
Blood pressure	Normal	81 (93.1)	1,125 (94.0)	0.81
	hypertension	6 (6.9)	72 (6.0)	
Mode of delivery	Normal vaginal delivery	37 (42.5)	661 (53.7)	**0**.**04**
	C-section	50 (57.5)	569 (46.3)	

PUC, pre-university course; GDM, gestational diabetes mellitus; PA, physical activity; SES, socioeconomic status; SSFT, sum of skinfold thickness.

Statistically significant findings (*p*-value < 0.05) are shown in bold.

Each unit increase in the social support score was associated with an increase in the PAL value (*β* = 1.65, *p* = 0.03) in the adjusted linear model. Similarly, each unit increase in the PAL value was associated with a decrease in prenatal depressive symptoms (*β* = −6.36, *p* < 0.001). Moreover, each unit increase in the PAL value was found to be associated with lower FBS and PPBS levels (*β* = −2.06, *p* = 0.04 and *β* = −0.99, *p* = 0.01) in the adjusted linear model (Table 3).

**Table 3:** Multivariable linear regression model depicting the relationship between an increase in physical activity level score and continuous pregnancy outcome variables in the MAASTHI birth cohort, South India (*N* = 1,317).

Variable	Unadjusted			Adjusted		
	*β* coefficient	95% CI	*p*-value	*β* coefficient	95% CI	*p*-value
Age	9.26	5.42 to 13.10	**<0**.**01**	2.60	−1.88 to 7.09	0.25
Gestational age	2.56	−0.39 to 5.51	0.08	1.63	−1.58 to 4.84	0.32
Income	0.002	0.00–0.004	0.11	0.001	−0.002 to 0.004	0.41
Prenatal depressive symptoms	−7.11	−10.21 to −4.01	**<0**.**001**	−6.36	−9.67 to −3.05	**<0**.**001**
Social support	2.60	1.14 to 4.05	**<0**.**001**	1.65	0.10 to 3.20	**0**.**03**
FBS	−2.09	−3.88 to −0.30	**0**.**02**	−2.06	−4.12 to −0.008	**0**.**04**
PPBS	−0.87	−1.58 to −0.16	**0**.**01**	−0.99	−1.80 to −0.18	**0**.**01**
SBP	1.78	0.30 to 3.25	**0**.**01**	1.17	−0.88 to 3.22	0.26
DBP	1.86	0.08 to 3.64	9.04	0.55	−1.87 to 2.98	0.65
Hemoglobin	−2.50	−16.45 to 11.44	0.72	−6.55	−20.92 to 7.81	0.37
Sum of skinfold thickness	1.86	0.76 to 2.96	0.001	1.86	−0.04 to 3.76	0.05
Weight	2.20	0.77 to 3.62	0.003	−0.18	−3.10 to 2.72	0.90
MUAC	6.58	2.27 to 10.90	0.003	−1.90	−11.07 to 7.27	0.68

CI, confidence interval; DBP, diastolic blood pressure; EPDS, Edinburgh Postnatal Depression Scale; FBS, fasting blood sugar; MUAC, mid-upper arm circumference; PPBS, postprandial blood sugar; SBP, systolic blood pressure.

Age, socioeconomic status, parity, pregnancy EPDS score, social support during pregnancy, blood pressure, blood sugar levels, and the sum of skinfold thickness during pregnancy have been adjusted for in the adjusted analysis. Statistically significant findings (*p*-value < 0.05) are shown in bold.

The odds of having elective C-section delivery were 4.32 times higher for the women who engaged in sedentary behavior during pregnancy compared to the women who engaged in a moderate level of activity [Odds Ratio (OR) = 4.32, CI: 2.62–7.11, *p* ≤ 0.01]. Furthermore, the women who engaged in sedentary behavior had higher odds of obesity as measured through SSFT (OR = 1.07, CI: 1.01–1.14, *p* = 0.02) and MUAC (OR = 1.04, CI: 1.0–1.09, *p* = 0.04). There was no significant relationship between sedentary behavior during pregnancy and other maternal outcomes such as GDM, prenatal depressive symptoms, and poor social support in the multivariable logistic regression analysis (Table 4).

**Table 4:** Multivariable logistic regression for the association between sedentary behavior during pregnancy and adverse pregnancy and delivery health outcomes (*N* = 1,317).

Variable	Category	OR	Standard error	95% CI	*p-*value
Delivery type	Vaginal delivery	1 (Ref)			
	Emergency C-section	0.27	0.04	0.19 to 0.38	<0.01
	Elective C-section	4.32	1.09	2.62 to 7.11	**<0**.**01**
Education	Illiterate	1 (Ref)			
	Primary school	0.145	0.12	0.02 to 0.78	0.025
	Middle school	0.165	0.13	0.03 to 0.79	0.025
	High school	0.11	0.08	0.02 to 0.51	<0.01
	PUC/diploma	0.09	0.07	0.02 to 0.46	<0.01
	Graduate	0.02	0.02	0.005 to 0.12	<0.01
	Postgraduate	0.007	0.008	0.001 to 0.07	<0.01
Socioeconomic status	Lower class	1 (Ref)			
	Upper-lower class	1.173	0.33	0.66 to 2.06	0.58
	Lower middle class	.816	0.23	0.46 to 1.44	0.48
	Upper middle class	1			
	Upper class	1			
Depressive symptoms	Absent	1 (Ref)			
	Present	1.001	0.01	0.97 to 1.03	0.94
Gestational diabetes	No	1 (Ref)			
	Yes	1.14	0.25	0.73 to 1.78	0.54
Sum of skinfold thickness	≤90th percentile	1 (Ref)			
	>90th percentile	1.07	0.03	1.01 to 1.14	**0**.**02**
Mid-upper arm circumference	≤90th percentile	1 (Ref)			
	>90th percentile	1.04	0.02	1.01 to 1.09	**0**.**04**
Social support score	>24	1 (Ref)			
	0–24	1.007	**0**.**007**	0.99 to 1.02	0.32

Adjusted for age, education, socioeconomic status, parity, depressive symptom score, social support, mid-upper arm circumference, parity, gestational diabetes mellitus, and the sum of skinfold thickness. Statistically significant findings (*p*-value < 0.05) are shown in bold.

## Discussion

We found that each unit increase in PAL during pregnancy was strongly associated with a decrease in the total score of prenatal depressive symptoms, fasting, and postprandial blood sugar level. In addition, we observed that the pregnant women who had good social support tended to engage in higher levels of PA. Conversely, sedentary behavior during pregnancy was associated with higher odds of being obese as measured through SSFT and MUAC and also undergoing an elective C-section delivery.

The findings of our study suggest that a moderate level of activity during pregnancy can contribute to reducing prenatal depressive symptoms. This is consistent with previous research, including a systematic review and meta-analysis by Daley et al. which found that PA interventions during pregnancy can moderately reduce depressive symptoms (44). Another meta-analysis by Liu et al. reported that higher levels of PA during pregnancy were associated with a lower risk of perinatal depressive symptoms (45). Our study adds to this existing evidence by using a well-validated measure of PA and demonstrating a dose-response relationship between PA and prenatal depressive symptoms. We also found a positive association between PA levels and social support scores, which is supported by previous research. For instance, a study by Bennetter et al. found that overall family support was associated with an increase in moderate to vigorous levels of PA (46). In addition, a systematic review found that individuals with greater social support for PA are more likely to engage in some form of PA (21). Overall, our study provides further evidence of the benefits of PA during pregnancy and highlights the importance of social support in promoting PA in an urban Indian population.

Several previous studies have investigated the relationship between sedentary behavior during pregnancy and the likelihood of C-section delivery. The evidence suggests that increased PA during pregnancy may increase the chances of normal delivery (47). A randomized controlled trial found that moderate-intensity PA during pregnancy was associated with a reduced frequency of C-section delivery (48). Our study supports these findings by demonstrating that pregnant women who engage in sedentary behavior had higher odds of elective C-section delivery, even after adjusting for potential confounders. However, few studies have explored the relationship between PA during pregnancy and adverse maternal outcomes in India. A study by Anjana et al. reported that GDM was significantly associated with sedentary behavior compared to women without GDM (8).

In this study, we found that the association between the continuous data of exposure (physical activity) and outcomes (depressive symptom score, social support score, FBS, and PPBS value) was significant while the categorized data for both the exposure and outcome based on predefined cutoffs did not show any significant association. This shows that using cutoffs to define two or more groups for exposure or outcome variables may tend to lose significant results compared to the use of continuous data in the analysis. However, this also suggests that the existing exposure cutoffs should be reviewed periodically.

Our research has significant implications for urban areas in India and other LMICs where the problem of adverse pregnancy and delivery outcomes is prevalent. In India, the high prevalence of C-section deliveries is associated with maternal and fetal morbidity and mortality. Our previous study revealed that babies born through elective C-section delivery may be at risk of obesity and reduced linear growth at 1 year of age (16). In addition, antenatal depressive symptoms during pregnancy were strongly associated with small for gestational age (SGA) babies (49). We also found that moderate levels of activity among pregnant women were linked to reducing postnatal depressive symptoms (20).

The Global Action Plan on Physical Activity emphasizes the importance of making PA a part of daily life for individuals of all ages (50). The WHO 2020 physical activity guidelines also stress the importance of reducing sedentary behavior throughout the lifespan (51). Despite the growing evidence of the benefits of physical activity during pregnancy, it has not been fully integrated into life course research (52). A recent review has highlighted the advantages of incorporating life course epidemiology into physical activity research (53). However, many studies have limited application of life course epidemiology concepts and do not use objective physical activity data. Our work is a valuable contribution to life course epidemiology by incorporating physical activity data.

Physical inactivity is becoming a major public health issue in LMICs. Encouraging physical activity during pregnancy could have significant benefits for both the mother and baby. Our study contributes to this evidence by using a large sample size and the study was conducted at public hospitals in an urban area of India. We used a PA questionnaire which was validated against an accelerometer and a 24-h activity diary in the study setting. We conducted biochemical tests at accredited laboratory facilities, taking both internal and external quality control measures. We also calibrated the anthropometric equipment every month according to prescribed guidelines. The relevance of our study extends beyond urban India to other LMICs where there is a high burden of adverse maternal health outcomes.

The questionnaires used to evaluate the level of PA were based on subjective data, which could lead to over-reporting of data. This questionnaire was validated in the general adult population but not validated specifically in pregnant women. Since there is no marked difference in activity among these two populations, a validated questionnaire among the adult population in the study setting was used in the study. To safeguard the collected data, the study ensured the privacy and anonymity of the collected data. The study did not find any pregnant women with vigorous levels of PA, therefore only sedentary and moderate levels were considered for analysis. The study found a low prevalence of sedentary behavior, which could have resulted in a smaller number of true positive cases. Finally, our analysis of data on physical activity, prenatal depressive symptoms, social support, and anthropometric characteristics was cross-sectional in nature.

## Conclusions

The findings of our study highlight the potential association between sedentary behavior during pregnancy and higher odds of maternal obesity, C-section delivery, higher blood glucose levels, and depressive symptoms. We also noted that good social support was associated with moderate-level activity. Promoting physical activity during pregnancy is an effective way to improve maternal health outcomes. Additional research could be required to assess the effectiveness of physical activity interventions in reducing adverse pregnancy and delivery outcomes. Healthcare providers and policymakers in India should consider promoting physical activity through a comprehensive approach in routine prenatal care and an effective strategy to improve social support for pregnant women is required.

## Data Availability

The raw data supporting the conclusions of this article will be made available by the authors, without undue reservation.
