# Supplementary material for: Relationship between physical activity during pregnancy and maternal health outcomes: evidence from the MAASTHI cohort study in Bengaluru, India
Source: Front Sports Act Living. 2025 Jan 30;7:1265929. doi: 10.3389/fspor.2025.1265929 (PMC11821958; doi:10.3389/fspor.2025.1265929)
Supplement: Supplementary file 1 [file Table1.docx]

**Supplementary file 1**

**The effect size for the physical activity being exposure and outcome being pregnancy and delivery health outcomes**

| **Outcome** | **Cohen's d** | **Effect-size r** |
| --- | --- | --- |
| Depressive symptoms | 0.11 | 0.05 |
| Social support | -0.09 | -0.04 |
| Gestational diabetes mellitus | 0.05 | 0.02 |
| Hypertension | 0.02 | 0.01 |
| Sum of skinfold thickness | 0.06 | 0.03 |
| Mid upper arm circumference | 0.03 | 0.01 |
| Delivery type | 0.04 | 0.02 |
